# Diagnosis of the Deadbeat

**Published:** 1909-07

**Authors:** E. S. McKee


### Diagnosis of the Deadbeat.

Would it not be delightful if we had tests to tell this evil as we have for blood, sputum, urine, feces? Deadbeat is an Americanism, and like a great many of them, is very expressive. A deadbeat is one who never pays, though often quite able to do so. He lives by evasions, and is an utterly dishonest fellow. Dead, here, intensifies the meaning of the word beat. One who is badly beat is spoken of as beat all to sticks. Beats who are very expert are described as, Would beat Caesar himself. Shakespeare tells us "Beat not the bones of the dead." Though absent or dead, simply look on your books and speak of the dear deadbeat, though a dead give away. A deadbeat in saloon parlance is said to mean a compound of ginger, soda and whisky, while in newspaper lingo it has quite a different meaning. The world is the deadbeat's country, and the inhabitants thereof his meat. Shakespeare seems to have had some advanced ideas on asepsis, for he says: "I'll beat thee, but I should infect my hands." A daisy deadbeat is the euphonious name applied to a swindler of the first water. The medical profession are the peculiar prey of this gentry. Speaking generally, colored people, prostitutes and gamblers are good pay only when they pay cash, though I have seen marked exceptions in each of these classes. People who hold unimproved Seattle real estate, undeveloped mining stock, great expectation of inheriting from an old relative, should be looked upon with suspicion. Inhabitants of Continental Europe will probably pay a small bill promptly when they first come over, but in time will learn the trick of their deadbeat neighbors. The second generation will dress better and pay worse. The physiognomy of the deadbeat is somewhat characteristic, though not pathognomonic. He has a bold, hail-fellow-well-met, indifferent air of effrontery. First, lead him on a recital of his former medical attendants, their mistakes and failures; then when you get home call them up and find how much they are out. I have often saved myself more chagrin by following this method. Given a good case of deadbeat, always prove your diagnosis by a post-mortem when possible.

A patient who shows an utter lack of consideration for the doctor and other patients should be regarded with suspicion. One who carefully counts and, when possible, saves the doctor's visits, generally intends to pay for the same, though he may want them at a reduced rate. Morphine and cocaine fiends usually lose all honor, and, of course, with the rest a willingness or desire to pay the doctor. They can be put down, generally, as sure to beat you if you trust them. The man who owes you and is able to pay you, but will not, is seldom your friend, but usually your enemy. He will try to ease his conscience, if any, by making himself and his acquaintances believe that you have done him harm instead of good, and that you do not deserve your money. Doctors ought to post each other as to deadbeats. If a doctor has been badly swindled by one of these wretches he should by all means let his neighbors know, so that they might protect themselves. Things are not always what they seem, and one may be mistaken in his diagnosis of the deadbeat ante-mortem as easy as that of any other disease. We sometimes get a respectable fee where we least expected it, and likewise lose one where we least expected. The diagnosis of the deadbeat is a scientific and necessary part of medicine. Let the doctor learn to diagnose the deadbeat and avoid him. Put his time and money to a better use; watch the business side of medicine more carefully; accumulate a little surplus, so that, along in the

50's or 60's, as he thinks best, he may be able to take life more easily, and in a different sense than he did in his early practice.

A vine-clad home on a quiet street,
An easy chair and slippered feet;
With wife of his youth, enough to eat,
And never a thought for the darned deadbeat.
—*E. S. McKee, in Gaillard's Southern Medicine.*

